# 390. Eravacycline combination therapy for severe, recurrent, or fulminant *Clostridioides difficile* infection

**DOI:** 10.1093/ofid/ofac492.468

**Published:** 2022-12-15

**Authors:** Austin R Morrison, Shaina Kwiatkowski, Mayur Ramesh, Rachel M Kenney

**Affiliations:** Moffitt Cancer Center, Tampa, Florida; Henry Ford Macomb Hospital, Detroit, Michigan; Henry Ford Hospital, Detroit, Michigan; Henry Ford Hospital, Detroit, Michigan

## Abstract

**Background:**

Eravacycline (ERV) is a fluorocycline with in vitro activity against *Clostridioides difficile* infection (CDI). The purpose of this study was to evaluate the usage of ERV in the management of CDI.

**Methods:**

IRB-approved, retrospective case series in a health system that added ERV to formulary in 9/2019. All patients between 9/2019 and 2/2020 treated with adjunctive ERV for > 24 hours for severe, recurrent, or fulminant CDI were included. Exclusion criteria: pregnant, age < 18 years. Primary outcome: all-cause mortality at 30 days (d) from start of ERV. Secondary outcomes: clinical cure, colectomy, and recurrence within 30 d. Data was reported using descriptive statistics and measures of central tendency.

**Results:**

14 patients included: severe (4, 29%), recurrent (4, 29%), and fulminant CDI (6, 43%) (table 1). Infectious diseases consult: 14/14, median time to consult 1 (1, 2) d. Surgery consult: 1 severe and 5 fulminant CDI cases, median time to consult 1 (1, 3) d. Prior to ERV initiation, 10 patients were on oral vancomycin (PO VAN) and intravenous metronidazole (IV MTZ), one was on PO VAN, two were on IV MTZ, and one was on no CDI therapy. After ERV was initiated, six patients were on ERV, PO VAN, and IV MTZ combination and eight patients were on ERV and PO VAN concurrently. The reason for using ERV was fulminant CDI (6, 42.8%), severe CDI (4, 29%), unable to tolerate other CDI medications (3, 21%), refractory CDI (3, 21%), and recurrent CDI (1, 7%). Time to eravacycline initiation 1.5 d (1, 3.75) with median duration of 6 d (4.5, 7.75). 30-day all-cause mortality 2 (14%), all were in-hospital; 1 (7%) hospice. Clinical cure occurred in 12 (86%). Two (14%) required colectomy; one received surgery on the same day of CDI diagnosis and ERV initiation and the other had surgery 4 days before ERV initiation. Two patients with recurrent CDI received fecal microbiota transplant outpatient, one of which also received bezlotoxumab. Zero recurrences and one readmission within 30 d.

**Table 1.**
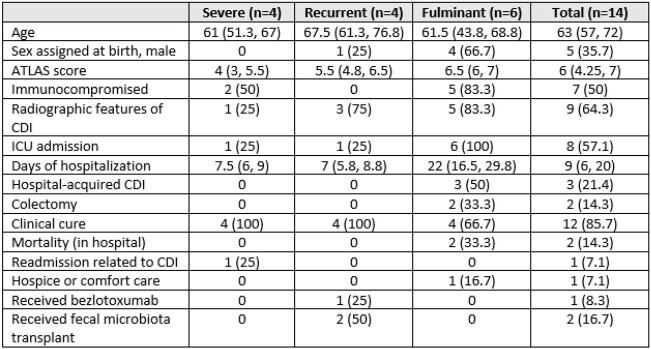
Patient demographics, severity, and clinical outcomes

**Conclusion:**

ERV appears to be a potential adjunctive therapy for severe, recurrent, or fulminant CDI. Prospective studies are needed to further investigate the safety and efficacy of ERV in serious CDI.

**Disclosures:**

**All Authors**: No reported disclosures.

